# Estimation of Carcass Tissue Composition from the Neck and Shoulder Composition in Growing Blackbelly Male Lambs

**DOI:** 10.3390/foods11101396

**Published:** 2022-05-12

**Authors:** Miguel A. Gastelum-Delgado, José Antonio Aguilar-Quiñonez, Carlos Arce-Recinos, Ricardo A. García-Herrera, Ulises Macías-Cruz, Héctor A. Lee-Rangel, Alvar A. Cruz-Tamayo, Juan C. Ángeles-Hernández, Einar Vargas-Bello-Pérez, Alfonso J. Chay-Canul

**Affiliations:** 1 Facultad de Agronomía, Universidad Autónoma de Sinaloa, km 17.5 Carretera Culiacán-El Dorado, Culiacán C.P. 80000, Sinaloa, Mexico; Miguel.angel.gastelum@uas.edu.mx (M.A.G.-D.); palomo_98@hotmail.com (J.A.A.-Q.); 2 División Académica de Ciencias Agropecuarias, Universidad Juárez Autónoma de Tabasco, Carretera Villahermosa-Teapa, km 25, R/A, La Huasteca 2ª Sección, Villahermosa C.P. 86280, Tabasco, Mexico; carlos.arce@colpos.mx (C.A.-R.); ricardo.garcia@ujat.mx (R.A.G.-H.); 3 Instituto de Ciencias Agrícolas, Universidad Autónoma de Baja California, Ejido Nuevo León S/N, Mexicali C.P. 21705, Baja California, Mexico; umacias@uabc.edu.mx; 4 Facultad de Agronomía y Veterinaria, Centro de Biociencias, Universidad Autónoma de San Luis Potosí, Tulancingo de Bravo C.P. 78000, San Luis Potosí, Mexico; hector.lee@uaslp.mx; 5 Facultad de Ciencias Agropecuarias, Universidad Autónoma de Campeche, Escárcega C.P. 24350, Campeche, Mexico; alalcruz@uacam.mx; 6 Instituto de Ciencias Agropecuarias, Universidad Autónoma del Estado de Hidalgo, Av. Universidad Km. 1, Tulancingo de Bravo C.P. 43600, Hidalgo, Mexico; juan_angeles@uaeh.edu.mx; 7 Department of Veterinary and Animal Sciences, Faculty of Health and Medical Sciences, University of Copenhagen, Grønnegårdsvej 3, DK-1870 Frederiksberg C, Denmark; 8 Department of Animal Sciences, School of Agriculture, Policy and Development, University of Reading, Earley Gate, P.O. Box 237, Reading RG6 6EU, UK

**Keywords:** carcass muscle, carcass fat, carcass bone, hair lambs

## Abstract

This study was designed to develop predictive equations estimating carcass tissue composition in growing Blackbelly male lambs using as predictor variables for tissue composition of wholesale cuts of low economic value (i.e., neck and shoulder). For that, 40 lambs with 29.9 ± 3.18 kg of body weight were slaughtered and then the left half carcasses were weighed and divided in wholesale cuts, which were dissected to record weights of fat, muscle, and bone from leg, loin, neck, rib, and shoulder. Total weights of muscle (CM), bone (CB) and fat (CF) in carcass were recorded by adding the weights of each tissue from cuts. The CM, CF and CB positively correlated (*p* < 0.05; 0.36 ≤ r ≤ 0.86), from moderate to high, with most of the shoulder tissue components, but it was less evident (*p* ≤ 0.05; 0.32≤ r ≤0.63) with the neck tissue composition. In fact, CM did not correlate with neck fat and bone weights. Final models explained (*p* < 0.01) 94, 92 and 88% of the variation observed for CM, CF and CB, respectively. Overall, results showed that prediction of carcass composition from shoulder (shoulder) tissue composition is a viable option over the more accurate method of analyzing the whole carcass.

## 1. Introduction

The economic viability is the central point to optimize production system decisions and profitability increases; and the carcass tissue composition provides valuable information for ensuring the viability of sheep meat production [1,2]. The proportions of muscle, fat, and bone in the carcass play an essential role in animal production, and this knowledge is fundamental for studying nutrition, physiology, and carcass quality [3]. The factors that affect the carcass characteristics and the carcass value of this type of animal have rarely been examined [4,5].

Although, it has been reported that the selling price would be improved if some carcass traits and several edible tissues were improved [4,5]. The prediction of energy retained from the diet by the animal requires a precise evaluation of the body composition, [6]. Several assays have reported that dissection of the entire carcass into muscle, fat, and bone is precise and used methods for predicting carcass tissue composition in small ruminants [3,7,8], but it is also costly, laborious, destructive [3], time-consuming, produces carcass losses and requires specialized staff [9,10]. Thus, indirect methods have been developed to estimate the carcass tissue composition, such it is the case of predictive equations to determine muscle, fat, and bone weights in sheep and goat carcasses using the tissue composition only from some anatomic regions (i.e., rib, leg, and shoulder) [10,11,12,13]. Rivera-Alegria et al. [2], using the neck to develop some predictive equations, reported a positive relationship between the neck and hot and cold carcass weights; this showed that the neck is a suitable predictor variable for carcass muscle weight and fat from hair sheep lambs.

Some hair sheep breeds such as Pelibuey and Blackbelly are usually used in the tropics. These are maternal breeds and not many reports are available on their carcass characteristics [9,10,14,15]; which are vital for promoting economic efficiency in these production systems [10]. Furthermore, indigenous breeds have a critical genetic and cultural value in these regions and are important for the economy of low-income farmers [1,10,14,16].

It is noteworthy saying that breed, diet, and age at slaughtering are the major factors affecting the composition of the small ruminant carcass [17]. In this sense, very few studies are carried out for predicting composition of the carcass in tropical hair sheep, to generate information that is important for decision-making in the tropical production systems of sheep [18]. Therefore, this study developed predictive equations for carcass tissue composition utilizing the neck and shoulder composition of growing Blackbelly male lambs.

## 2. Materials and Methods

### 2.1. Experimental Site and Animals

Lambs were handled in compliance with the guidelines and regulations for ethical animal experimentation of the División Académica de Ciencias Agropecuarias, Universidad Juárez Autónoma de Tabasco (ID project PFI: UJAT-DACA-2015-IA-02).

The experiment was carried out at the Sheep Integration Center of the Southeast (17° 78″ N, 92° 96″ W; 10 masl). Forty growing Blackbelly male lambs, with average body weight (BW) of 29.1 ± 2.88 kg (±SD) and from 5 to 8 months of age, were used. Lambs were housed in raised-slatted floor cages with a group feeding system (ten animals per cage). The diet was a total mixed ration (80:20 concentrate to forage ratio) containing ground corn, soybean meal, star grass hay, vitamins and minerals premix. The diet had a crude protein level of 15% DM and 12 MJ of metabolizable energy [19].

Lambs were fasted for 24 h to record shrunk BW (SBW) and then slaughtered according to the Mexican Official Standard NOM-033-SAG/ZOO-2014. All bodies were bled, skinned, eviscerated, and then the carcasses were cooled at 4 °C for 24 h to record cold carcass weight (CCW). Subsequently, carcasses were split longitudinally with a band saw to obtain the individual weight of the left half carcass, which then was divided into five wholesale cuts (i.e., leg, loin, neck, rib, and shoulder). Finality, all muscle mass obtained by cutting was combined to record carcass muscle weight (CM); a similar procedure was applied to record carcass fat (CF) and bone (CB) weights. Particularly, weights of neck and shoulder muscle, fat, and bone were individually recorded to be considered as predictor variables.

### 2.2. Data Analyses

Database exploration began with the detection of outliers using a boxplot to visualize the median and the spread of the data. Although linear regression is reasonably robust against violation of normality of data [20], the assumption of normality was assessed plotting the probability distribution and calculating the kurtosis and skewness of all variables. The descriptive analysis was performed using the “describe” function of psych package [21]. The next step was exploring the relationship between dependent and explicative variables, which included a graphical exploration through a multi-panel scatterplot and a pairwise Pearson’s correlation analysis. Lastly, results were displayed in a correlogram plot using the GGally package [22].

Model selection: The final database included three dependent variables (CM, CF, and CB) and eight independent variables or predictors (SW = Shoulder weight, SM = Shoulder muscle, SF = Shoulder fat, SB = Shoulder bone, NW = Neck weight, NM = Neck muscle, NF = Neck fat and NB = Neck bone). The procedure to compare the performance of the different multiple regression models for choosing the best one was carried out by implementing an exhaustive search through a stepwise sequential replacement method combining forward and backward selection. The criteria used during the stepwise procedure to select the best models were Schwartz’s information criterion (BIC) and adjusted determination coefficient (r2 adj). The stepwise process added and pruned explanatory variables in models to reach a balance between model simplicity (parsimony) and predictive performance. The models to each dependent variable were chosen, and their goodness of fit was evaluated. The Akaike´s Information Criterion (AIC), Schwartz’s information criterion (BIC), adjusted determination coefficient (r^2^adj), and root mean square error (RMSE) were the criteria de goodness of fit. Models with the lowest AIC and BIC, RMSE, and highest r^2^adj were defined as the best models.

To improve the accuracy of estimations of each model derived from the stepwise process, a multicollinearity test was performed. The multicollinearity in multiple regressions models was explored using the Variance Inflation Factor (VIF). Calculation of VIF and plots was carried out using the “JTOOLSs” package [23].

The performance of models was not only evaluated with their fit of data; therefore, the best model must be also parsimonious. The choice between a simple and very complex model implies that the complex model provides a much better fit if the data is set in order to justify the increase of complexity. In the current work to compare the fits of two models, we used the ANOVA function with regression models as two separated arguments. To this test, if the value of *p*-value is lower to 0.05 indicated that the estimations of compared models are different, which means that the more complex model is significantly better than the simpler model.

### 2.3. Model Validation

The predictive ability of models selected was evaluated using k-fold validation methods, with k = 10 (k = 10). The k groups were randomly made, and this was repeated three times. The performance of the fitted model in predicting the actual observations was evaluated using the RMSE, R^2^, and mean absolute error (MAE). The lowest values of RMSPE and MAE indicated the best predictions. For validation, RMSPE and MAE were the averages of cross-validation. The k- folds validation was implemented in the “Classification and Regression Training” package [24]. This package allows comparing numerous multivariate calibration models under a unified framework.

## 3. Results

The lambs had a SBW between 23.2 and 34.9 kg with a CCW ranging from 7.96 to 17.01 kg. The average conformation of the carcasses was 9.28 ± 1.52 kg of muscle, 3.06 ± 0.41 kg of bone, and 1.27 ± 0.42 kg of fat (Table 1). The average weights for shoulder and neck were 1.30 ± 0.20 and 0.68 ± 0.17 kg, respectively, and their tissue compositions evidenced a higher content of muscle, followed by bone and fat (Table 1).

**Figure 1:**
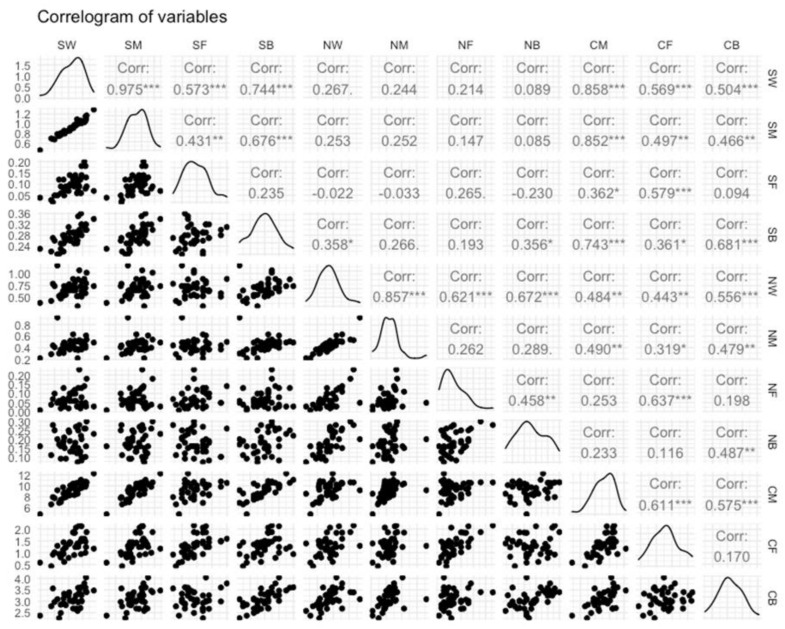
Graphical analysis of the input and output variables. Scatterplots, distributions, and correlation coefficients of shoulder weight (SW), shoulder muscle (SM), shoulder fat (SF), shoulder bone (SB), neck weight (NW), neck muscle (NM), neck fat (NF), neck bone (NB), carcass muscle (CM), carcass fat (CF), carcass bone (CB). *** *p* < 0.001; ** *p* < 0.01; * *p* < 0.05.

Pearson correlation coefficient results are shown in Figure 1. With exception of the correlation between CB and SF (*p* > 0.05), all carcass tissue components positively correlated (*p* < 0.001) with overall weight (0.50 ≤ r ≤ 0.86) and tissue component weights (0.36 ≤ r ≤ 0.85) of shoulder. With regard to neck, all carcass tissue components had positive correlation (*p* < 0.001; 0.32 ≤ r ≤ 0.56) with NW and NM, while CF correlated (*p* < 0.001) only with NF (r = 0.64), and CB with NB (r = 0.49).

Results of developed equations and their validation are shown in Table 2, Table 3, Table 4 and Table 5. Three equations by carcass tissue components (i.e., muscle, fat, and bone) were developed (*p* < 0.05) with similar R^2^ values to each other within each component (Table 2). Thus, the equations (Eq.) to predict the amount of carcass muscle tissue explained between 81 and 83% of the variation observed in the dependent variable (Eq. 1 to 3). However, Eq. 2 was the best because it had lower values for AIC and BIC without multicollinearity problems (≤ 2.56) among predictor variables (i.e., SM, SB, and NM; Table 3). In addition, the cross-validation test showed that this model had the highest R^2^ (0.89 vs. ≤0.85) combined with the lowest values in the error estimators (RMSPE = 0.64 vs. ≥0.67, and MAE = 0.56 vs. 0.61) compared to other models (Table 4). In fact, the parsimony analysis shows that Eq. 2 is better (*p* = 0.02) than Eq. 1 but similar (*p* = 0.42) to Eq. 3; however, Eq. 2 is less complex based on a number of predictors than Eq. 3, whereby these findings confirm Eq. 2 as the best for predicting CM (Table 5).

For carcass fat tissue, eq. 4, 5 and 6 explained between 62 and 63% of the variation observed in this dependent variable, and none of them showed multicollinearity problems (VIF ≤ 2.56). Eq. 4 and 5 had lower AIC (~11.29 vs. 11.95) and BIC (~20.58 vs. 23.77) values while cross-validation results showed that Eq. 6 is better due to its lower RMSPE and MAE values, and higher r^2^. The parsimony analysis showed that the three models are optimal (*p* ≥ 0.31) to be used, although Eq. 4 could be better because the number of predictor variables (n = 3 vs. 4 o 5) is less than in the other equations. Overall, although Eq. 6 had slightly higher AIC and BIC values, this equation seems to be the ideal one as it has the best goodness of fit (lower MSPE = 0.055 vs. ≥0.057) and prediction capacity (cross-validation results). So, Eq. 6 included as predictors to SM, SF, NW, NF, and NB.

To predict amount of carcass bone tissue, Eq. 7, 8 and 9 were developed (*p* < 0.05) and explained 55, 57 and 56% of the variation observed in CB. Eq. 9 was discarded because it had the highest AIC and BIC values, likewise it did not meet the collinearity assumption. Compared to Eq. 7, the Eq. 8 showed slightly better goodness of fit (lower MSPE = 0.059 vs. 0.063, and AIC = 10.34 vs. 11.04) and prediction accuracy (lower RMSPE = 0.31 vs. 0.32, and MAE = 0.24 vs. 0.25), but 4% lower prediction capacity (r^2^ = 0.54 vs. 0.50). Finally, Eq. 7 and 8 could be used to predict BC (*p* = 0.12) as suggested by the parsimony test.

## 4. Discussion

Hair sheep play a vital economic role in tropical regions of the American continent due to their prolificacy, hardiness, parasite resistance, and adaptability to different environmental conditions [18]. However, few studies have been conducted to predict carcass composition, an aspect of vital importance for determining retained energy and energy requirements for maintenance [6,25]. Therefore, this study proposes equations for predicting CM, CF, and CB with high accuracy.

Proportions of muscle, bone and total fat were 68.1, 22.4, and 9.34%, respectively. CM in this study was higher than those reported for hair lambs Pelibuey [26], and Katahdin crossed with meat breeds (Vázquez et al., 2011) but the values corresponded to back and shoulder blade tissue composition, respectively, while CF was lower in the present study. Tshabalala et al. [27] reported proportions of carcass tissue (dissected) in Dorper breed lambs with values of 75.4% for CM, 10.4 for CF, and 14.46 for CB, which differs from the results obtained in this study. Resentment, Kecici et al. [28] reported in Kivircik lambs carcass tissue proportions with 49.6% for CM, 21.87% for CF, and 21. 2% for CB differs from the results obtained in this study. It is to be expected that the proportion of muscle, fat, and bone will vary among studies because factors such as breed, nutritional level of the diet, age, and slaughter weight influence the carcass composition of small ruminants [17].

Keçici et al. [28] mentioned that half-carcass tissue composition could be predicted using some joints such as neck (muscle %), ribs (bone and subcutaneous fat %), flank (intermuscular fat %), and hind limb (total fat %). Though, none of the carcass joints alone were adequate for predicting all tissue composition.

The data used to determine regression equations must comply with the assumptions of normality, linearity, and non-multicollinearity. In this sense, the variables used to determine CM, CF and CB had low to moderate correlation between them in a range of 0.32 to 0.72 (Table 2). Correlations >0.80 between independent variables produce biased estimates due to multicollinearity problems [29]. On the other hand, decision regression three could give several benefits, as no assumption is required on the distribution of explanatory variables. Also, these should be applied to the dependent variables including categorical, numerical, and survival data, and it is not influenced by high correlations among independent variables. For that, the dependent variables to explain the model are the most important, and the insignificant variables are excluded [8].

In this study, the equations described had high predictive accuracy for the carcass composition of Blackbelly lambs. The r^2^ for CM ranged from 0.80 to 0.94, the values for CF ranged from 0.77 to 0.92, while the lowest values were recorded in the prediction of BC (r^2^ = 0.55−0.88). This study’s coefficient of determination values agrees with previous studies in lambs. For example, in Blackbelly sheep, Canton et al. [9] reported that CM and CB could be predicted by thorax muscle and bone (r^2^ = 0.86 and 0.83, respectively). For the same breed, Garcia-Osorio et al. [30] reported that leg and shoulder muscle weight explained 90 and 96% of the variation in CM, while thorax and shoulder bone weight explained 89 and 84% of the variation in BC in 56-day-old twin- and single-born lambs, respectively. However, both studies reported no equations for CF prediction. Recently, predictive equations for CM, CF, and CB in Kivircik lambs, the shoulder muscle weight was used to predict CM with an r^2^ of 0.64. Also, for the prediction of CB, the hind limb bone weight was used, however, a low coefficient of determination (0.45) was observed; while for prediction of CF the hind limb total fat weight was used as a predictor, and an r^2^ of 0.75 was obtained [28].

The leg, shoulder, and rib dissected from the carcass of Blackbelly lambs were the anatomical regions that most accurately predict carcass composition (CM, CF, and CB), which is consistent with studies reported in small ruminants [11,12,13]. The integration of these three anatomical regions (leg, rib, and shoulder) gave greater accuracy to the equations for the prediction of CM and CB, while for CF, it was leg, rib, and neck. In this sense, Miguélez et al. [31] observed that the least predictive accuracy for carcass tissue components was from neck tissues. Moreover, except for the neck, Kempster et al. [32] mentioned that the composition of any joint was suitable for carcass composition predicitions.

## 5. Conclusions

Total weights of muscle, bone, and fat in carcass were positively correlated with shoulder components but not with the neck tissue composition in Blackbelly sheep. The models obtained in the current study reached from 88 to 94% of the variation observed in the carcass tissue composition. Overall, results showed that prediction of carcass composition from shoulder (shoulder) tissue composition is a viable option over the more accurate method of analyzing the whole carcass.

## Figures and Tables

**Table 1:** Descriptive analyses of the data measured in live animals (*n* = 40) fattening Blackbelly sheep.

Item	Description	Mean	SD	Min	Max	Skew	Kurtois
SW	Shoulder weight (kg)	1.30	0.20	0.74	1.70	−0.50	−0.22
SM	Shoulder muscle (kg)	0.92	0.15	0.46	1.29	−0.37	0.39
SF	Shoulder fat (kg)	0.09	0.04	0.02	0.20	0.45	−0.23
SB	Shoulder bone (kg)	0.27	0.03	0.21	0.36	0.05	−0.50
NW	Neck weight (kg)	0.68	0.17	0.32	1.17	0.51	0.32
NM	Neck muscle (kg)	0.43	0.12	0.22	0.92	1.35	4.32
NF	Neck fat (kg)	0.06	0.04	0.00	0.23	1.23	1.42
NB	Neck bone (kg)	0.17	0.06	0.07	0.30	0.15	−1.07
CM	Carcass muscle (kg)	9.28	1.52	4.83	12.26	−0.59	0.32
CF	Carcass fat (kg)	1.27	0.42	0.43	2.15	0.19	−0.58
CB	Carcass bone (kg)	3.06	0.41	2.18	4.04	0.09	−0.39

SD, standard deviation; Min, minimum; Max, maximum; Skew, skewness.

**Table 2:** Predictive regression equations for carcass tissue composition using the neck and shoulder tissue traits as predictors in Blackbelly male lambs (*n* = 40).

ID	Model	Adj. R^2^	MSPE	AIC	BIC
1	= 0.29(0.69) + 5.61(0.51) × W + 3.63(0.87) × NM	0.81	0.37	82.67	89.42
2	= −0.36(0.76) + 5.62(0.83) × SM + 10.49(3.62) × SB + 3.26(0.83) × NM	0.83	0.33	79.27	87.72
3	= −0.40(0.76) + 5.33(0.91) × SM + 2.16(2.67) × SF + 10.68(3.65) × SB + 3.36(0.85) × NM	0.82	0.32	80.53	90.66
	Carcass fat (CF)				
4	= −0.05(0.24) + 0.75(0.29) × SM + 3.31(1.15) × SF + 4.52(0.91) × NF	0.62	0.061	11.38	19.83
5	= −0.17(0.25) + 0.62(0.30) × SM + 3.68(1.16) × SF + 0.51(0.37) × NM + 4.15(0.93) × NF	0.62	0.057	11.20	21.33
6	= −0.06(0.27) + 3.09(0.31) × SM + 3.09(1.29) × SF + 0.55(0.37) × NW + 4.17(1.22) × NF − 1.41(0.94) × NB	0.63	0.055	11.95	23.77
	Carcass bone (CB)				
7	= 0.91(0.32) + 5.98(1.22) × SB + 0.78(0.25) × NW	0.55	0.063	11.04	17.81
8	= 0.84(0.32) + 5.82(1.19) × SB + 1.08(0.31) × NW − 1.74(1.09) × NF	0.57	0.059	10.34	18.79
9	= 0.87(0.32) + 5.67(1.21) × SB + 1.66(0.77) × NW − 0.73(0.90) × NM − 2.56(1.50) × NF	0.56	0.057	11.61	21.73

Shoulder weight (SW), shoulder muscle (SM), shoulder fat (SF), shoulder bone (SB), neck weight (NW), neck muscle (NM), neck fat (NF), neck bone (NB), carcass muscle (CM), carcass fat (CF), carcass bone (CB), adjusted determination coefficient (r^2^adj), mean square error (MSPE), Akaike´s Information Criterion (AIC) and Schwartz’s information criterion (BIC).

**Table 3:** Evaluation of multicollinearity of proposed models using Variance Inflation Factor (VIF).

Model	SW	SM	SF	SB	NW	NM	NF	NB
1	1.06					1.06		
2		1.86		1.88		1.09		
3		2.21	1.27	1.88		1.11		
4		1.23	1.29				1.08	
5		1.35	1.37			1.19	1.17	
6		1.41	1.68		2.56		2.03	2.08
7				1.15	1.15			
8				1.15	1.80		1.63	
9				1.18	11.14	7.01	3.0	

Shoulder weight (SW), shoulder muscle (SM), shoulder fat (SF), shoulder bone (SB), neck weight (NW), neck muscle (NM), neck fat (NF), neck bone (NB). VIF values between 5 and 10 indicates that the regression coefficients are poorly estimates due to multicollinearity.

**Table 4:** Proposed models using k-Fold cross-validation.

ID	Predictors	RMSPE	r^2^	MAE	RMSPE (SD)	R^2^ (SD)	MAE (SD)
Carcass muscle (CM)							
1	SW, NM	0.67	0.82	0.61	0.27	0.17	0.23
2	SM, SB, NM	0.64	0.89	0.56	0.23	0.09	0.21
3	SM, SF, SB, NM	0.68	0.85	0.61	0.26	0.15	0.24
Carcass fat (CF)							
4	SM, SF, NF	0.28	0.51	0.24	0.10	0.30	0.069
5	SM, SF, NM, NF	0.29	0.55	0.25	0.10	0.29	0.061
6	SM, SF, NW, NF, NM	0.27	0.62	0.22	0.08	0.28	0.043
Carcass bone (CB)							
7	SB, NW	0.32	0.54	0.25	0.19	0.37	0.15
8	SB, NW, NF	0.31	0.50	0.24	0.14	0.36	0.11
9	SB, NW, NM, NF	0.32	0.52	0.25	0.12	0.37	0.11

Shoulder weight (SW), shoulder muscle (SM), shoulder fat (SF), shoulder bone (SB), neck weight (NW), neck muscle (NM), neck fat (NF), neck bone (NB), carcass muscle (CM), carcass fat (CF), carcass bone (CB), adjusted determination coefficient (r^2^), root mean square error (RMSPE), mean absolute error (MAE) and standard deviation of r^2^, RMSPE and MAE.

**Table 5:** Assessment of parsimony of the proposed models.

Comparison	Df ^1^	*p*-Value ^2^
	Carcass muscle (CM)	
Model 1 vs. model 2	1	0.02
Model 1 vs. model 3	2	0.07
Model 2 vs. model 3	1	0.42
	Carcass fat (CF)	
Model 4 vs. model 5	1	0.16
Model 4 vs. model 6	2	0.23
Model 5 vs. model 6	1	0.31
	Carcass bone (CB)	
Model 7 vs. model 8	1	0.12
Model 7 vs. model 9	2	0.22
Model 8 vs. model 9	1	0.42

^1^ Df, an indicator of additional parameters of a more complex model. ^2^ *p*-value lower to 0.05 indicating that a more complex model is significantly better than the simpler model.

## Data Availability

The data presented in this study are available on request from the corresponding author.
